# Effectiveness and safety of a microcrystalline tyrosine‐adjuvanted *Dermatophagoides pteronyssinus* allergoid immunotherapy in adult patients with allergic asthma and rhinitis: A real‐life prospective observational study

**DOI:** 10.1002/iid3.585

**Published:** 2022-04-19

**Authors:** Clara Padró, Diego Gutiérrez, Francisco Moreno, Antonio Parra, Manuel J. Rial, Ramón Lleonart, Carla Torán‐Barona, José L. Justicia, Albert Roger

**Affiliations:** ^1^ Allergy Section Hospital Universitari Germans Trias i Pujol Badalona Spain; ^2^ Clínica Médico Asistencial Virgen del Rosario Algeciras Spain; ^3^ CM ASISA Dr. Lobatón Cádiz Spain; ^4^ Allergy Department Complexo Hospitalario Universitario A Coruña A Coruña Spain; ^5^ Allergy Unit Hospital Universitari de Bellvitge L'Hospitalet de Llobregat Spain; ^6^ Allergy Therapeutics Ibérica Sant Joan Despí Spain

**Keywords:** allergen‐specific immunotherapy, allergic asthma, allergoid, *Dermatophagoides pteronyssinus*, microcrystalline tyrosine, real‐life clinical practice

## Abstract

**Introduction:**

Although clinical trials have shown the efficacy and safety of allergen‐specific immunotherapy (AIT) in the treatment of allergic asthma, there is a need for real‐life studies. We aimed to assess the effectiveness and safety of a microcrystalline tyrosine‐adjuvanted *Dermatophagoides pteronyssinus* allergoid (Acarovac Plus®) in patients with house dust mite (HDM)‐induced allergic asthma in a real‐life study.

**Methods:**

A subanalysis of a multicenter, prospective, observational, real‐life study. Patients with rhinitis and allergic asthma caused by HDMs were assessed before AIT with Acarovac Plus® and at 6 and 12 months after this treatment. Assessment parameters were percentage of days with asthma symptoms, percentage of days on asthma medication, classification of asthma according to Spanish guidelines for the management of asthma, asthma‐related quality of life (quality of life in adults with asthma questionnaire [QLAAQ]), perception of symptoms (visual analog scale [VAS]), and treatment satisfaction (treatment satisfaction questionnaire for medication [TSQM]). Safety was assessed by the number and severity of adverse reactions.

**Results:**

This subanalysis included 55 patients. Treatment with Acarovac Plus® showed significant differences in the analyzed variables when the baseline visit was compared with the 12‐month visit: reduction of the mean (SD) percentage of days with asthma symptoms (23.9 [9.2] vs. 5.1 [12.8]; *p* = .002), of the mean [SD] percentage of days on asthma medication (67.6 [42.9] vs. 45.1 [46.8]; *p* = .002), and of the percentage of patients with persistent asthma (78.2% vs. 38.9%; *p* = .009). Acarovac Plus® significantly improved asthma‐related quality of life, as shown by a decrease of 1.39 points in QLAAQ score at 12 months (*p* < .001), and in the subjective perception of symptoms on the VAS (−3.50, *p* < .0001). Patients showed high treatment satisfaction according to the TSQM, and it was well tolerated. No serious adverse events were reported.

**Conclusions:**

Acarovac Plus® was effective and safe for the treatment of patients with HDM‐induced allergic asthma in a real‐life study.

## Graphical abstract

In this multicenter, prospective, observational, real‐life study, we assessed the effectiveness and safety of allergen‐specific immunotherapy with a microcrystalline tyrosine‐adjuvanted *Dermatophagoides pteronyssinus* allergoid (Acarovac Plus®) in patients with house dust mite‐induced allergic asthma. At 6 and 12 months of treatment, Acarovac Plus® showed a significant clinical improvement assessed by reduction of days with symptoms and need for medication and by improvement in disease severity according to Spanish guidelines for the management of asthma classification and asthma‐related quality of life. All of the above was associated with a good safety profile and a high level of patient satisfaction.

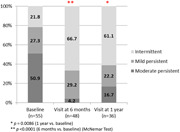

**Abbreviations**
AIT: allergen‐specific immunotherapy
AQLQ: asthma quality of life questionnaire
DF: *Dermatophagoides farinae*
DP: *Dermatophagoides pteronyssinus*
FEV1: forced expiratory volume in 1 s
GEMA: Spanish guidelines for the management of asthma
HDM: house dust mites
MCT: microcrystalline tyrosine
QLAAQ: quality of life in adults with asthma questionnaire
TSQM: treatment satisfaction questionnaire for medication
VAS: visual analog scale

## 1 INTRODUCTION

Asthma is a prevalent disease—in 2017, about 272 million people were estimated to be affected worldwide—which entails a major burden in terms of health and quality of life.^1^ In Spain, its prevalence is estimated to be 5%.^2^ House dust mites (HDM), such as *Dermatophagoides pteronyssinus* (DP) or *Dermatophagoides farinae* (DF), are the most significant source of allergens that have been associated with allergic asthma in the world,^3^ and the second most significant source of sensitization in Spain.^4^ HDM avoidance measures are not always feasible and, in many cases, despite these measures, patients with rhinitis and allergic asthma still have symptoms. Allergen‐specific immunotherapy (AIT) is usually indicated in patients with asthma symptoms that cannot be controlled with avoidance measures and usual symptomatic treatment, and in patients who do not tolerate or adhere to treatment regimens.^5^

AIT has been shown to be a safe and efficacious treatment for allergic asthma in several clinical trials.^6^, ^7^ In patients with asthma and allergic sensitization, AIT has been associated with decreased symptom scores and medication requirements, and with an improvement in allergen‐specific and nonspecific bronchial hyperresponsiveness.^7^, ^8^, ^9^ Therefore, the European Academy of Allergy and Clinical Immunology (EAACI) recommends integrating HDM AIT in the general treatment of allergic asthma.^7^

Acarovac Plus® is a specific AIT with purified mite allergen extracts (*DP, DF, Blomia tropicalis*, or *Lepidoglyphus destructor*) modified into allergoids by treating them with glutaraldehyde and associated with microcrystalline tyrosine (MCT). Previous observational studies have shown the clinical and immunological efficacy and tolerability of this product.^10^, ^11^ However, these studies were all single‐center and included a reduced number of patients. Therefore, more real‐life practice studies are needed to confirm these data. Recently, the real‐life practice, multicenter ALL‐ACA‐2014‐01 study has shown that Acarovac Plus® is an effective and well‐tolerated treatment in adult patients with allergic rhinitis, with or without asthma, which reduces allergic symptoms and the need for symptomatic medication while increasing patients' quality of life.^12^ The objective of this subanalysis was to assess the effectiveness and safety of Acarovac Plus® in patients with HDM‐induced allergic asthma, based on the data obtained in the abovementioned real‐life clinical practice study.

## 2 MATERIAL AND METHODS

### 2.1 Study design and population

ALL‐ACA‐2014‐01 was an observational, prospective, multicenter real‐world study conducted at 10 sites in Spain, all of them with a high allergenic load set by mites. The study included adult patients aged 18–65 years old, of both genders, diagnosed with allergic rhinitis with or without asthma sensitized to the HDM *DP* who were prescribed subcutaneous AIT (Acarovac Plus® DP 100%) according to standard clinical practice guidelines. The diagnosis was based on the patients' clinical history and physical examination.^12^ The present work is a subanalysis of the abovementioned study, included data from adult patients with both allergic rhinitis and asthma, who were treated with Acarovac Plus® for one year.

Other inclusion criteria were as follows: clinical symptoms of rhinitis and asthma caused by allergic sensitization to DP for at least 1 year before study initiation and for whom, in the investigator's opinion, treatment with specific immunotherapy was indicated; skin prick test result ≥3 mm in diameter for DP in the previous 12 months; positive result for a specific immunoglobulin E (IgE) to DP of at least Class 2 (greater than 0.7 kU/L measured by ImmunoCAP system; Thermo Fisher Scientific) at baseline; DP‐monosensitized or polysensitized patients, as long as the other sensitizations were not considered clinically relevant by the investigator; the specialist prescribed treatment with Acarovac Plus® 100% DP. Patients with severe persistent asthma, uncontrolled disease, forced expiratory volume in 1 s (FEV1) <70%, or in need of systemic corticosteroid therapy within 8 weeks before treatment started were excluded.

This study was approved by the Ethics Committee of the Germans Trias i Pujol University Hospital (reference: EPA‐14‐023), and complied with the main ethical guidelines in accordance with the Declaration of Helsinki. Informed consent was obtained from all the patients enrolled in the study.

### 2.2 Study medication

Acarovac Plus® (Allergy Therapeutics) is an injectable suspension for subcutaneous administration containing a purified allergen extract of *DP* modified into an allergoid by treatment with glutaraldehyde, and adjuvanted with MCT. The treatment regimen consisted of an initial (or up‐dosing) phase and a maintenance phase. The initial phase consisted of the administration of one 0.05 ml dose, followed by increasing doses of 0.1, 0.3, and 0.5 ml administered at weekly or biweekly intervals. When the dose of 0.5 ml was reached, the maintenance phase started, consisting of the administration of a 0.5 ml dose every 6 weeks until the end of treatment. The total duration of the follow‐up phase was 13 months. Assessments were conducted at the baseline visit (before treatment start), at 6 months after starting treatment, and at the final visit, 1 year after treatment start. Before and after administering the study medication, peak expiratory flow was measured.

### 2.3 Study variables

The assessed parameters were: the percentage of days with asthma symptoms, the percentage of days on medication, the classification of asthma disease according to the Spanish guidelines for the management of asthma (GEMA)^2^ (intermittent, mild persistent, moderate persistent, severe persistent), scores reported in the quality of life in adults with asthma questionnaire (QLAAQ),^13^ intensity of symptoms perceived by patients using a visual analog scale (VAS), patient satisfaction with the medication based on the treatment satisfaction questionnaire for medication (TSQM),^14^ and treatment‐related adverse reactions. All parameters were assessed 4 weeks before the treatment started and before each study visit.

Symptoms and the use of medication during the study were followed up using a diary that patients filled out every day at home for 4 weeks before each study visit. Asthma symptoms (cough, wheezing, and dyspnea), as well as use of medication (maintenance and rescue treatments), were collected.

QLAAQ^13^ was used to assess the asthma‐related quality of life, with reference to the 4 weeks before completion of the questionnaire, including four dimensions (dyspnea, mood disorders, social disorders, and health issues). The total score of the questionnaire was adjusted to a 0–10 scale, where higher values indicated a larger impact of asthma on quality of life. On the VAS, patients rated their symptoms from 0 (“no symptoms”) to 10 (“the worst possible symptoms”) by placing a mark on a straight line with 0 on one end and 10 on the other.

Patient satisfaction with the administered treatment was measured using TSQM‐14,^14^ which included four assessment dimensions (effectiveness, adverse effects, convenience, and overall satisfaction). The score of each dimension was adapted to a 0–100 scale, with a higher value representing a higher level of satisfaction.

All adverse reactions related to the administration of the product were collected and classified as local or systemic adverse reactions according to the EAACI.^15^

### 2.4 Statistical analysis

Categorical variables were described as relative frequencies (%), whereas continuous variables were expressed as the mean and standard deviation.

To compare quantitative variables between the different visits, the nonparametric Wilcoxon test was used. Qualitative data were analyzed using the *χ* ^2^ test or the McNemar test. All analyses were performed using the SAS statistical package, version 9.4.

## 3 RESULTS

### 3.1 Study population

In the ALL‐ACA‐2014‐01 study, a total of 141 patients were recruited, of which 118 (83.7%) met all inclusion criteria and none of the exclusion criteria, and were thus considered assessable. The sample was comprised of 56.8% of women, and the mean (SD) age was 33.6 (9.5) years.^12^ The present subanalysis included only the 55 (46.6%) patients of this population who had asthma (Figure S1).

The mean duration of the disease from diagnosis was 14.1 (SD 18.8) years. At baseline, 47 (85.5%) of them were using asthma medication: 36 (76.6%) were using maintenance treatment and 37 (78.7%) rescue treatment. The mean FEV1 value for these patients recorded at baseline was 91.1% (SD 12.9%, range 70–131). On the basis of GEMA classification criteria for asthma,^2^ at the baseline visit, 21.8% of patients had intermittent asthma, 27.3% had mild persistent asthma, and 50.9% had moderate persistent asthma.

### 3.2 Effectiveness data

**Figure 1:**
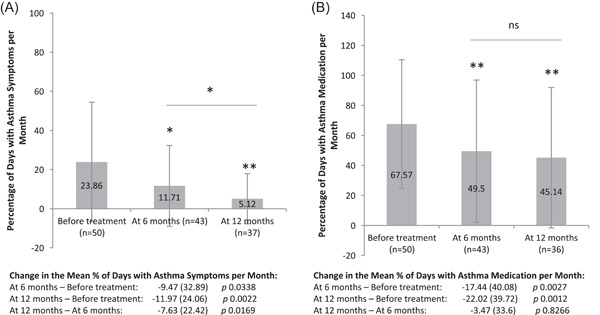
Change in the mean percentage of days with asthma symptoms per month (A) and in the mean percentage of days of use of asthma medication per month (B). *p < .05; **p < .005 (Wilcoxon test [nonparametric paired test]). ns, nonsignificant

During the period before treatment started, 28% of asthmatic patients were asymptomatic. This percentage increased to 44.2% at 6 months after treatment start with AIT (*p* = .1083) and to 75.7% at 1 year after treatment start (*p* = .0002 vs. the visit before treatment; *p* = .0027 vs. the 6‐month visit). The mean days with symptoms in the 4 weeks before each visit was 6.68 (SD 8.56) before treatment start, 3.28 (SD 5.78) at 6 months of treatment, and 1.43 (SD 3.59) 1 year after treatment start. Statistically significant differences of −2.65 (SD 9.21) days with symptoms (*p* = .0226) were observed between the period before treatment start and at 6‐month follow‐up, and of −3.35 (SD 6.74) days with symptoms (*p* = .0022) between the period before treatment start and at 12‐month follow‐up. The mean percentage of days with asthma symptoms per month was 23.86% in the period before treatment start, and decreased to 11.71% at the 6‐month follow‐up, and to 5.12% at the 12‐month follow‐up, with statistically significant differences between the baseline visit and at 6 months of treatment (*p* = .0338), between the baseline visit and at 1 year of treatment (*p* = .0022), and between 6 months and 1 year of treatment (*p* = .0169) (Figure 1A).

Regarding the use of asthma medication, 18% of patients did not use any medication in the period before treatment started. At the follow‐up visits, after starting AIT, this percentage increased to 27.9% at 6 months (*p* = .0253) and 38.9% at 1 year of treatment (*p* = .0114). The mean days requiring the use of asthma medication were 18.92 (SD 12.02) before treatment start, 13.86 (SD 13.27) at 6 months of treatment, and 12.64 (SD 13.10) 1 year after treatment start. Statistically significant differences of −4.88 (SD 11.22) days were observed between the baseline visit and at the 6‐month follow‐up (*p* = .0027), and of −6.17 (SD 11.12) days between the baseline visit and at 12‐month follow‐up (*p* = .0012). The mean percentage of days on asthma medication per month was 67.57% before treatment start, 49.50% at 6 months of treatment, and 45.14% 1 year after treatment start. Statistically significant differences were observed, with a decrease of 17.44% days between the baseline visit and the 6‐month visit (*p* = .0027) and a decrease of 22.02% days between the 6‐month visit and 1 year of treatment (*p* = .0012) (Figure 1B).

**Figure 2:**
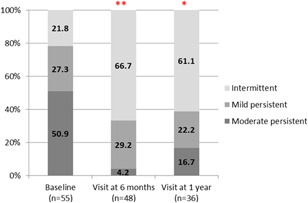
Classification of asthma severity according to Spanish guidelines for the management of asthma. Percentage of patients with asthma classified as intermittent, mild persistent, moderate persistent, and severe persistent at the baseline visit, at 6 months, and at 1 year of treatment. **p < .0001, 6 months versus baseline; *p* = .0086, 1 year versus baseline (Mcnemar test)

According to asthma severity data based on the GEMA guidelines^2^ (Figure 2), the percentage of patients with asthma classified as persistent (mild or moderate) was 78.2% at the baseline visit, decreasing to 35.2% at 6 months and 38.9% at 1 year of treatment, with statistically significant differences between the baseline and the 6‐month visits (*p* < .0001), and between the baseline and the 12‐month visits (*p* = .0086). Analyzed from another perspective, the percentage of patients with intermittent asthma increased from 21.8% at the baseline visit to 66.7% at the 6‐month visit, and to 61.1% at 1 year of treatment.

During the assessment of the asthma‐related quality of life based on QLAAQ,^13^ statistically significant differences were observed in all the dimensions of the questionnaire and throughout the study compared with the baseline visit. A mean decrease in the total score was recorded of 1.72 (SD 2.21) from the baseline visit to the 6‐month follow‐up (*p* < .0001) and of 1.39 (SD 2.09) from the baseline visit to the 12‐month follow‐up (*p* < .0001). No significant differences were observed between the 6‐month follow‐up visit and 1 year after treatment start (Table 1).

**Table 1:** Quality of life in adults with asthma questionnaire (QLAAQ) score

	*n*	Mean (SD)	*P* a
Dyspnea (0–20 scale)			
Baseline	52	7.12 (5.33)	
At 6 months	41	3.02 (3.24)	
At 12 months	34	3.09 (3.76)	
Change in dyspnea			
Visit at 6 months—baseline	41	−3.90 (5.39)	<.0001
Visit at 12 months—baseline	34	−3.29 (4.56)	<.0001
Visit at 12 months—visit at 6 months	32	0.25 (3.23)	.9504
Mood disorders (0–20 scale)			
Baseline	51	7.04 (5.80)	
At 6 months	42	2.19 (2.94)	
At 12 months	33	2.97 (3.84)	
Change in mood disorders			
Visit at 6 months—baseline	41	−4.98 (5.03)	<.0001
Visit at 12 months—baseline	33	−3.91 (5.21)	<.0001
Visit at 12 months—visit at 6 months	32	1.00 (3.01)	.1144
Social disorders (0–28 scale)			
Baseline	48	6.48 (7.43)	
At 6 months	41	3.12 (4.99)	
At 12 months	33	3.58 (5.63)	
Change in social disorders			
Visit at 6 months—baseline	38	−3.74 (6.67)	.0004
Visit at 12 months—baseline	31	−2.45 (6.59)	.0082
Visit at 12 months—visit at 6 months	31	0.65 (5.07)	.9615
Health issues (0–12 scale)			
Baseline	51	3.82 (3.59)	
At 6 months	43	1.88 (2.28)	
At 12 months	33	1.85 (2.60)	
Change in health issues			
Visit at 6 months—baseline	42	−2.24 (3.19)	<.0001
Visit at 12 months—baseline	33	−2.00 (3.16)	.0005
Visit at 12 months—visit at 6 months	33	−0.12 (2.78)	.8868
QLAAQ: Total score (0–80 scale)			
Baseline	48	24.21 (20.11)	
At 6 months	40	9.85 (10.99)	
At 12 months	33	11.58 (13.94)	
Change in QLAAQ: Total score (0–80 scale)			
Visit at 6 months—baseline	37	−13.78 (17.67)	<.0001
Visit at 12 months—baseline	31	−11.10 (16.75)	<.0001
Visit at 12 months—visit at 6 months	30	1.53 (12.16)	.6335
AQLQ: Total score (0–10 scale)			
Baseline	48	3.03 (2.51)	
At 6 months	40	1.23 (1.37)	
At 12 months	33	1.45 (1.74)	
Change in QLAAQ: Total score (0–10 scale)			
Visit at 6 months—baseline	37	−1.72 (2.21)	<.0001
Visit at 12 months—baseline	31	−1.39 (2.09)	<.0001
Visit at 12 months—visit at 6 months	30	0.19 (1.52)	.6335

^a^ Wilcoxon test (nonparametric paired test).

Patients' VAS assessment of symptom intensity showed significant differences. The mean score obtained at the baseline visit was 6.4 (SD 2.38), while at the 6‐month follow‐up visit, the score was 3.5 (SD 2.49) (*p* < .0001), and at 12 months, 2.9 (SD 2.53) (*p* < .0001).

Regarding subjective satisfaction with the treatment received, assessed using TSQM‐14,^14^ a mean score for each dimension was observed at the 6‐month visit: 72.6 (SD 14.4) for effectiveness; 84.5 (SD 10.1) for adverse effects; 77.0 (SD 11.9) for convenience; and 76.9 (SD 13.5) for overall satisfaction. At the final 12‐month visit, the mean score for each dimension was 74.7 (SD 14.7) for effectiveness; 90.0 (SD 3.5) for adverse effects; 77.8 (SD 11.5) for convenience; and 78.6 (SD 14.6) for overall satisfaction. There were no statistically significant differences in treatment assessment between the 6‐ and 12‐month follow‐up visits.

### 3.3 Safety outcomes

Thirteen (23.6%) patients had local mild and transitory adverse reactions at the injection site. Only one (1.8%) patient‐reported systemic reactions on two occasions (general malaise and headache), both classified as Grade 0 based on EAACI guidelines.^15^ No asthmatic attacks triggered by the administration of the treatment were recorded. No serious adverse effects were reported, and no adrenaline was required.

## 4 DISCUSSION

The results of this study in adult patients with allergic rhinitis and asthma showed a clear and significant clinical improvement of their asthmatic condition both after 6 months and 1 year of treatment with Acarovac Plus® as measured by a decrease in days with symptoms and the use of asthma medication, as well as an improvement in the severity of the disease according to GEMA.^2^ At the same time, a significant improvement was observed in these patients' quality of life—as measured through the QLAAQ questionnaire^13^—and a decreased subjective perception of their symptoms on a VAS scale after 6 and 12 months of treatment. Furthermore, patients reported a high level of satisfaction with the treatment received, as measured through TSQM.^14^ The treatment also proved to be safe and well‐tolerated.

Patients with HDM‐induced allergic asthma are at high risk of developing wheezing, decreased pulmonary function, and bronchial hyperresponsiveness. Therefore, treating asthma symptoms is important to prevent exacerbations and progression of the disease to more severe stages.^2^, ^16^ However, most studies assessing HDM AIT are conducted in patients with allergic rhinitis, with limited studies analyzing this treatment in patients with allergic asthma.^7^

Acarovac Plus® is a commercially available subcutaneous AIT based on an HDM extract modified into an allergoid and MCT adsorbed. MCT is a biodegradable adjuvant with high adsorption^17^ properties and a 48‐h half‐life.^18^ This adjuvant also shows great immunogenic capacity, with increased T helper type 1 (interferon and interleukin 10 [IL‐10]) cytokine production and decreased IgE formation.^18^ These characteristics make this adjuvant optimal to modulate and enhance immune response, thus avoiding the use of aluminum hydroxide—which may induce Th2 responses—and the potential long‐term accumulation thereof.^19^ Our studies were aligned with previous trials that have shown clinical and immunological efficacy, as well as tolerability of Acarovac Plus®.^10^, ^11^, ^20^

The effectiveness of subcutaneous AIT for the treatment of patients with allergic asthma has been described in the literature for years,^6^ both in adult and pediatric patients.^21^ In the same way, the effectiveness of AIT has been shown in the specific treatment of HDM‐induced allergy in patients with allergic asthma, being the only treatment method that, aside from reducing symptoms and the use of medication, has the potential to alter the course of the disease.^22^ However, the only parameters used in daily clinical practice to measure the effectiveness of AIT are clinical, and there is no universally accepted measurement method.^23^ The Cochrane meta‐analysis of 2010 highlighted the wide variety of parameters found in the literature to assess AIT‐related asthma improvement.^24^ The review concluded that AIT may significantly reduce asthma symptoms, but that there is a significant disparity in the symptom scores used and in the different ways of defining the clinical condition. In contrast, the methods used to assess the reduction of the medication required to treat asthma were significantly homogeneous, a finding that is clinically useful, since it is one of the main objectives of AIT. That is why, the reduction of medication was one of the main parameters analyzed in this study to assess the effectiveness of treatment, in addition to the percentage of days with symptoms, the classification of the severity of the asthmatic disease according to GEMA,^2^ and specific questionnaires to assess patients' quality of life, symptom perception, and treatment satisfaction.

Other studies have also assessed the effectiveness of AIT with an extract of *DP* in patients with rhinitis and/or allergic asthma.^25^, ^26^, ^27^ A study conducted in Spain by Tabar et al.^27^ also found a significant clinical improvement of asthma at 1 year of treatment, as presented in our results, although a direct comparison of the results cannot be made due to the different assessment methods used. Regarding clinical improvement based on a VAS, the abovementioned study confirmed an improvement of 21% at 1 year of treatment versus 54.7% observed in our study.

On the other hand, our study recorded a significant improvement in terms of use of medication, with 38.9% of patients not requiring any rescue asthma treatment after one year of treatment. However, in the study conducted by Tabar et al., no significant decrease in the use of asthma medication after 1 year of treatment was observed which, the authors argued, could have been due to the fact that, during the study, patients were allowed to continue their background treatment to keep the disease under control and prevent potential adverse reactions, which means that it was more difficult to find variations in symptoms and rescue treatments, since patients were stable at baseline. Tabar et al.^28^ published a follow‐up of this study and found that, at 3 years posttreatment, 70% of patients did not need any medication and were asymptomatic. The fact that the percentage described in our study was not as high might be due to the lower duration of treatment (1 year vs. 3 years).

Another way to determine the effectiveness of AIT is by assessing the quality of life improvement through specific questionnaires (e.g., asthma quality of life questionnaire [AQLQ]^29^, ^30^ and QLAAQ^13^). An observational, multicenter Polish study^31^ assessed AQLQ score improvement after 3 years of AIT in 101 patients with allergic asthma, and a mean score increase of 0.84 was observed. In the study by Tabar et al.^28^ mentioned above, AQLQ scores in asthma patients showed a mean increase of 1.5 points. In our study, we analyzed asthma impact on quality of life based on the QLAAQ questionnaire, in which a mean decrease of 1.39 points was observed after only one year of treatment.

Multiple studies have shown both the efficacy and safety of AIT for the treatment of rhinitis as well as allergic asthma; therefore, several guidelines recommend the use of subcutaneous AIT for the treatment of HDM‐induced allergic asthma both in adults^7^, ^32^ and in the pediatric population.^7^, ^33^, ^34^

In Spain, Moreno et al.^35^ have conducted an observational, prospective multicenter study on the safety of immunotherapy with different sources of allergens and different commercial products for the treatment of both allergic rhinitis and asthma. Of a total of 17,526 injections analyzed, 0.6% were related with local reactions (corresponding to 11.9% of patients), and only 0.3% were related with systemic reactions (corresponding to 3.7% of patients) of Grades 2–3 (resolved with treatment). No anaphylactic shocks or fatal reactions were recorded. However, a higher frequency of systemic reactions was in fact recorded in asthmatic versus nonasthmatic patients (0.41% vs. 0.06%; *p* < .001), as well as in patients treated with AIT for mite allergy compared with those receiving immunotherapy with pollen extracts (0.40% vs. 0.15%; *p* < .01), although these conditions were not shown to be risk factors associated with systemic reactions caused by AIT when analyzed using multivariable logistic analysis. The abovementioned safety outcomes were slightly different from the results obtained in our study, where 23.6% of patients reported local reactions and only 1.8% reported systemic reactions (all mild and not requiring medication). On the other hand, our rate of systemic adverse reactions was similar to that described in the real‐life clinical practice EASSI study (2.1%).^36^

Some strong points of the design of this analysis are that it is prospective and multicenter in nature, studying multiple variables and that it was conducted in real‐life conditions, which has allowed us to assess the effect of Acarovac Plus® in the general population, facilitating the assessment of results in daily clinical practice conditions, and contributing to the increasing importance of real‐life studies. On the other hand, it had certain limitations that should be mentioned. Since this subanalysis included only patients with asthma, the population sample was very small, accounting for less than half the population of the whole study (patients with rhinitis and asthma), thus reducing its power. It has as well the limitations of a noninterventional study in a real‐life setting, and it must be taken into account that there was no control group in this study. Although the effectiveness variables assessed in this subanalysis were subjective (i.e., symptoms, use of medication, and disease perception) and could be influenced by a placebo effect, in the ALL‐ACA‐2014‐01 study, other objective variables were assessed and significantly improved, such as nasal provocation test. These are objective outcomes that are not depending on the patient or investigator's opinions.^12^ Furthermore, other analyses of this study have been recently published, providing evidence of increased levels of DP‐sIgG4, Der p 1 sIgE, and IL‐10 as mechanisms of immune tolerance induced by AIT specific for HDMs.^37^ Another limitation of the study was that data were collected using a diary that patients filled out daily. Studies with self‐reporting data are very common in epidemiologic and medical research, although several biases may occur, such as social desirability and recall bias.^38^ Even with these limitations, we believe that this methodology was adequate for the objectives of the study. Likewise, numerous studies in the literature evaluate patients with asthma using self‐reported diaries.^39^, ^40^, ^41^ Nevertheless, caution should be exercised when assessing results, since it would be necessary to conduct clinical trials with mites and a control group (placebo). Furthermore, other mite species should be analyzed in addition to DP. Finally, we should also highlight the need to conduct the same type of study with other populations (e.g., the pediatric population) in which rhinitis and allergic asthma are also highly prevalent conditions.

## 5 CONCLUSION

Patients with allergic bronchial asthma caused by allergy to *Dermatophagoides* treated with Acarovac Plus® (100% DP) in a real‐life practice study showed a significant clinical improvement assessed by the reduction of days with symptoms and of the need for medication, as well as an improvement in the severity of the disease according to GEMA.^2^ This study also recorded a significant improvement in asthma‐related quality of life both at 6 months and at 12 months of treatment. And all of the above was associated with good tolerance of the treatment and a high level of patient satisfaction.

## CONFLICT OF INTERESTS

Clara Padró has received support for attendance and participation in courses and conferences from Allergy Therapeutics Ibérica, during the conduct of the study and outside the submitted work. Albert Roger has received grants and personal fees from Allergy Therapeutics, during the conduct of the study; personal fees from LetiPharm, Hal, Roxall, Stallergenes, Allergopharma, and Diater, outside the submitted work. Manuel J. Rial has received personal fees from Astra Zéneca, GsK, Leti pharma, and Allergy Therapeutics, outside the submitted work. Carla Torán‐Barona and José L. Justicia are full employees of Allergy Therapeutics. Diego Gutiérrez, Francisco Moreno, Antonio Parra, and Ramón Lleonart have no conflict of interests.

## AUTHOR CONTRIBUTIONS

Clara Padró, José L. Justicia, and Albert Roger conceptualized and designed the study. Clara Padró, Albert Roger, Manuel J. Rial, Carla Torán‐Barona, Diego Gutiérrez, Francisco Moreno, Antonio Parra, Ramón Lleonart, and José L. Justicia contributed to data analysis and interpretation of the findings. Clara Padró, Albert Roger, Manuel J. Rial, Carla Torán‐Barona, Diego Gutiérrez, Francisco Moreno, Antonio Parra, Ramón Lleonart, and José L. Justicia drafted the manuscript and reviewed and approved the final manuscript. All authors meet the conditions of the International Committee of Medical Journal Editors regarding authorship.

## Supporting information

Supporting information.

## Data Availability

The data underlying this article will be shared at reasonable request to the corresponding author.
