# Supplementary material for: Effectiveness and safety of a microcrystalline tyrosine‐adjuvanted Dermatophagoides pteronyssinus allergoid immunotherapy in adult patients with allergic asthma and rhinitis: A real‐life prospective observational study
Source: Immun Inflamm Dis. 2022 Apr 19;10(5):e585. doi: 10.1002/iid3.585 (PMC9017636; doi:10.1002/iid3.585)
Supplement: Supplementary file 1 — Supporting information. [file IID3-10-e585-s001.docx]

# Effectiveness and Safety of a MicroCrystalline Tyrosine-Adjuvanted *Dermatophagoides pteronyssinus* Allergoid Immunotherapy in Adult Patients with Allergic Asthma and Rhinitis: A Real-Life Prospective Observational Study

# Supplementary Material


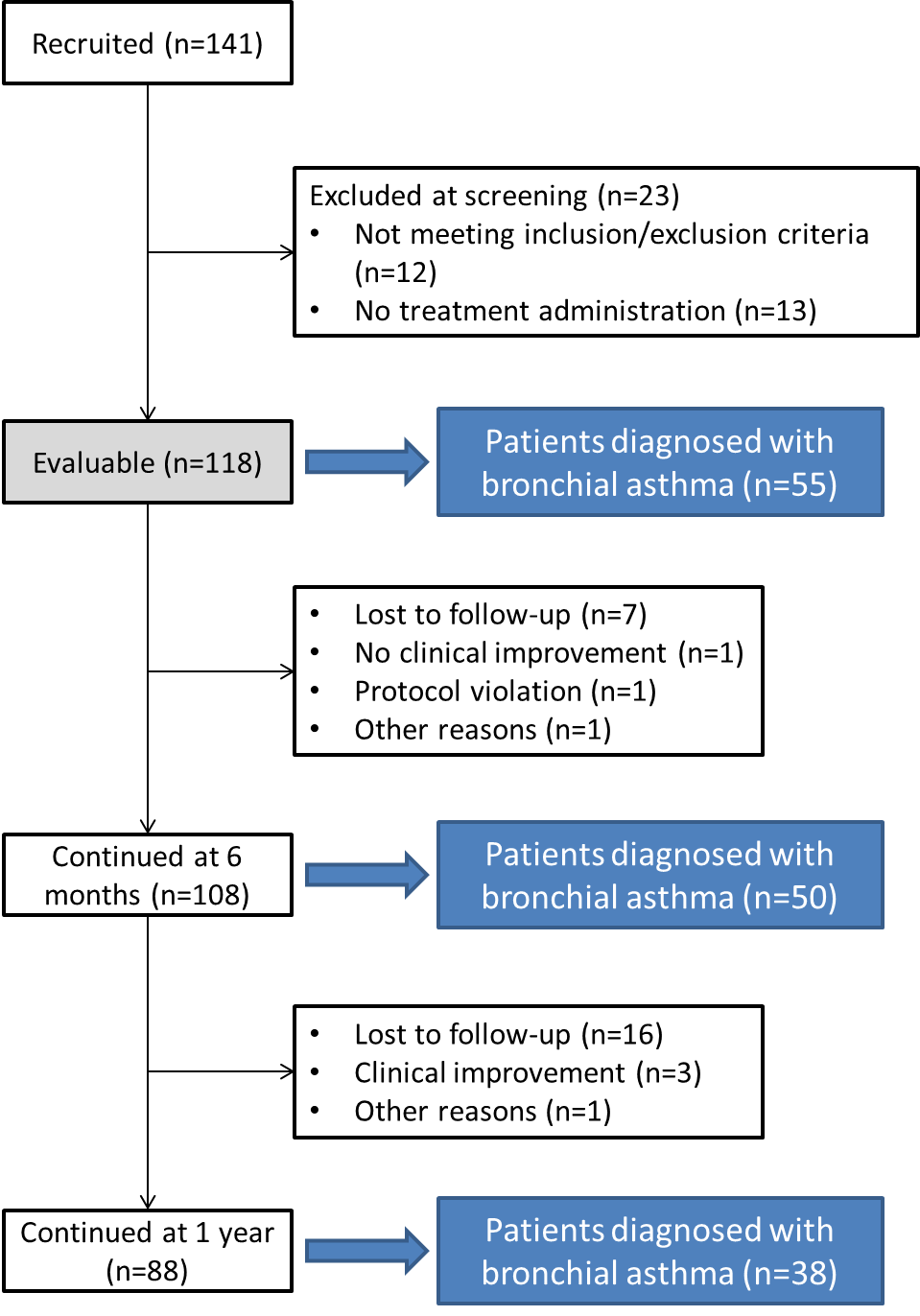


**Supplementary Figure 1.** Flow diagram of participants
